# Differences in quality of care, mortality, suicidal behavior, and readmissions among migrants and Danish-born inpatients with major depressive disorder

**DOI:** 10.1192/j.eurpsy.2022.2329

**Published:** 2022-10-21

**Authors:** Søren Valgreen Knudsen, Jan Brink Valentin, Marie Norredam, Poul Videbech, Jan Mainz, Søren Paaske Johnsen

**Affiliations:** 1 Department of Health Research, Danish Center for Clinical Health Services Research (DACS), Department of Clinical Medicine, Aalborg University, 9000 Aalborg, Denmark; 2 Psychiatry Region North Jutland, 9000 Aalborg, Denmark; 3 Danish Research Centre for Migration, Ethnicity, and Health (MESU), Department of Public Health, University of Copenhagen, 1415 Copenhagen, Denmark; 4 Center for Neuropsychiatric Research, Mental Health Center Glostrup, University of Copenhagen, 2600 Glostrup, Denmark; 5 Department of Community Mental Health, Haifa University, Haifa, Israel

**Keywords:** inequality, migrants health, mortality, quality of care, suicide

## Abstract

**Background:**

The increasing global migration has made migrants’ health a pertinent topic. This article aimed to examine whether migrants were less likely than Danish-born residents to receive guideline recommended care when hospitalized for major depressive disorder (MDD) and potential differences in clinical outcomes, including all-cause mortality, suicidal behavior, and readmissions during 1-year follow-up after first-time admission.

**Methods:**

A national cohort study was performed, including all adult MDD inpatients at mental care units in the period 2011–2017. Migrants and two migrant subgroups (non-Western and Western) were compared with Danish-born patients. Quality of care was examined using multivariable Poisson and linear regression models. Clinical outcomes were examined using Cox proportional hazards regression analysis.

**Results:**

Migrant-status was associated with a non-significantly lower chance of receiving high-quality care (relative risk [RR] = 0.93, confidence interval [CI] 0.86:1.01) and lower readmission rates for depression (hazard rate ratio [HR] = 0.93, CI 0.86:1.01), and significantly higher all-cause mortality (HR = 1.55, CI 1.19:2.01) and lower all-cause readmission rate (HR = 0.88, CI 0.83:0.94). No clear association was found regarding suicidal behavior. While associations were comparable for migrant subgroups regarding readmission, the associations with low quality of care and of all-cause mortality appeared strongest among Western migrants.

**Conclusions:**

Among inpatients with MDD in a universal tax-financed healthcare system, being a migrant was associated with a potential lower quality of in-hospital care and worse clinical outcomes. These results warrant further investigation to clarify the underlying explanation for these inequalities and to develop and test interventions to ensure better quality of care and clinical outcomes for migrant patients.

## Introduction

The increasing global migration has been a central force in the demographic changes of the European population. As of January 1, 2021, a total of 37.5 million persons born outside the European Union (EU) were living in the EU, corresponding to 8.4% of the entire population [1]. This considerable migration has made migrants’ health a pertinent topic [2]. The topic has become even more urgent with a large number of refugees as a result of Russia’s invasion of Ukraine in early 2022. Migrants represent a vulnerable population regarding mental disorders, since they may be exposed to risk factors before, during, and after migration [3]. A recent systematic review and meta-analysis found that one in four migrants and one in three refugees and asylum seekers suffer from depression globally [4]. Several studies have demonstrated an increased prevalence of depression among migrants compared with local born European populations [5–7], even though the prevalence varies according to the reason for migration and sex [7, 8]. Recent systematic reviews have shown that especially refugees are at higher risk of depression and other mental disorders than the average population [9, 10].

Although migrants are disproportionately affected by depression, little is known about the quality of inpatient depression care and clinical outcomes among migrants compared with the local born population. The aim of this national study was to examine whether migrants were less likely than Danish-born residents to receive clinical guidelines recommended depression care when hospitalized for depression. Furthermore, potential differences in clinical outcomes, including mortality, suicidal behavior, and readmissions were examined during 1-year follow-up between migrants and Danish-born patients.

## Methods

### Study design

A national register-based cohort study was performed based on data from national registers in Denmark (5.8 million inhabitants). The cohort consisted of inpatients admitted in the period 2011–2017 with major depressive disorder (MDD) as the primary diagnosis. The Danish public healthcare system is mainly tax-funded with universal health coverage to ensure, in principle, free and equal access to hospital care for all Danish residents. Since no private mental care units exist, all patients with MDD are admitted and treated in public hospitals. A unique personal identifier, assigned to all residents, was used to register the use of services as well as the quality of care in national registers [11]. This identifier was used to retrieve and merge individual data from the different registers.

### Data sources

The Danish Depression Database is a nationwide clinical quality registry [12]. It was established in 2011 and contains information on the quality of care, admission and discharge dates for all adult patients (≥18 years) with permanent residence in Denmark, admitted at psychiatric hospitals with a primary diagnosis of MDD (International Classification of Diseases [ICD]-10: F32, F33, F34.1, and F06.32 including all subcodes) [12]. It is mandatory by law for all Danish mental care units to report data to the registry [12].

Data from the Danish National Patient Registry was used to identify readmissions with a primary diagnosis of MDD, all-cause readmission, and suicide attempts. Danish National Patient Registry includes patients treated at all non-psychiatric hospitals from 1977 and patients from psychiatric hospitals from 1995 and onwards [13].

The patients’ vital status was obtained through the Danish Civil Registration System. Established in 1968, it is a national register containing basic personal data of anyone with a social security number in Denmark [14]. The causes of death, including suicide, were obtained through the Register of Causes of Death, which has existed in its current form since 1970 [15].

### Study population

**Figure 1.**
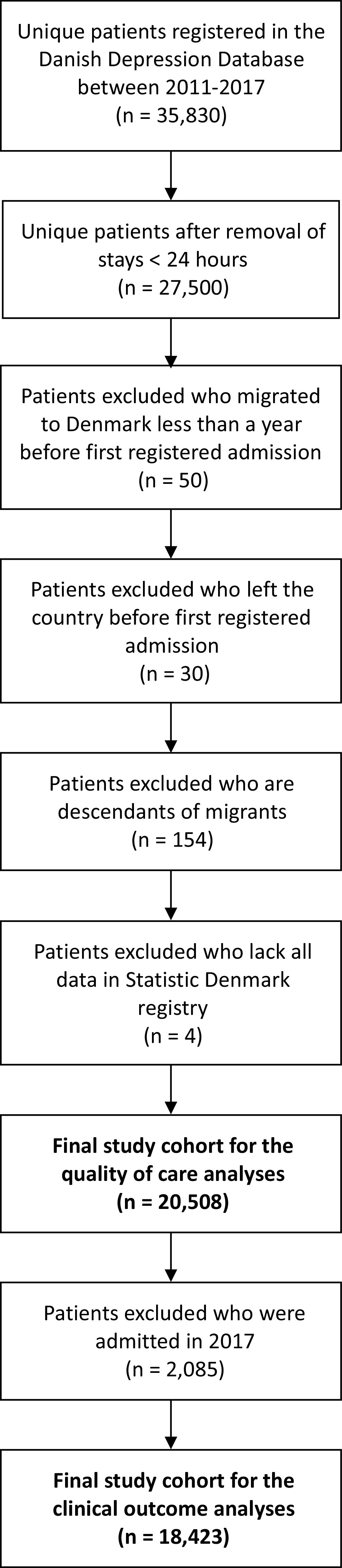
Flow chart of study population selection.

The study population was identified through the Danish Depression Database and included the first documented admission of adult (≥18 years) inpatients in the period 2011–2017 with MDD as the primary diagnosis (see Figure 1 for study population selection). Stays shorter than 24 h were excluded, since data collection in the Danish Depression Database only includes those who stay longer than 24 h. If multiple hospital contacts occurred within periods of up to 4 days, they were considered to reflect the same admission. Some of these combined admissions contained contacts shorter than 24 h. However, the combined admission was excluded if all contacts were <24 h. In total, 20,750 individual first-time documented admissions between 2011 and 2017 were identified. Among these, subjects were excluded if they had migrated to Denmark within 1 year before (*n* = 50) or had left the country before the first registered admission in the Danish Depression Database (*n* = 30). This was done to ensure complete data on the covariates and clinical outcomes. Four subjects lacked all data in the Statistic Denmark registry and were therefore also excluded. Finally, descendants of migrants were excluded since they represent a subgroup with characteristics of both migrants and the native population (*n* = 154). In Statistics Denmark, descendants are defined as people born in Denmark to parents, neither of whom was born in Denmark and has Danish citizenship. The final study cohort included 20,508 patients. In the analyses of the clinical outcomes, follow-up time was set to 1 year. Thus, patients admitted in 2017 were omitted from these analyses (*n* = 2,085).

### Exposure

According to Statistics Denmark, migrants are individuals born abroad to parents born abroad who are not Danish citizens [16]. Data from the Danish Depression Database were linked to the Population Register to obtain individual data on country of birth as well as immigration and emigration dates. The population was classified as migrants and non-migrants (Danish-born). Migrants were further subclassified into “Western” or “non-Western” according to their country of birth using the categorization of countries made by Statistics Denmark [16]. Due to the limited number of migrants in the Danish Depression Database, subclassification of the migrants into separate countries was not possible. This meant that heterogeneous groups in terms of migration experience, trauma exposure, socioeconomic status, and culture were grouped together. Asylum seekers who have not yet received a residence permit in Denmark are not provided a unique personal identifier and were therefore not included in the study.

### Outcomes

#### Quality of care

Quality of care was assessed by nine evidence-based performance measures obtained from the Danish Depression Database reflecting recommendations from national clinical guidelines [12] (see Table 1).

**Table 1.** Performance measures of quality of care in the Danish Depression Database for inpatients.

Performance measures		Definition
1	Examination by psychiatrist	The patient’s psychopathological assessment was performed by a specialist in psychiatry within 7 days after admittance to the hospital ward
2	Somatically examined	Neurological examination, relevant laboratory tests, and other examinations within 2 days of admittance
3	Assessment by social worker	Assessment of the need for acute or longer-term support, such as help with changing housing, financial help with purchasing medicine, educational guidance, rehabilitation, and application for disability benefits
4	HAM-D17 assessment (in)	Initial assessment using HAM-D17 within 7 days of admittance
5	HAM-D17 assessment (out)	Assessment using HAM-D17 at discharge from hospital
6	Suicide risk assessment (in)	Using structured interview at admittance
7	Suicide risk assessment (out)	Clinician’s assessment of the patient’s risk of suicide when discharge from hospital is planned
8	Contact with relatives	Staff have established or tried to establish contact with the patient’s relatives during hospitalization
9	Psychiatric aftercare	Before discharge, an agreement has been made on a specific time and place for follow-up care after discharge in an outpatient clinic or at the general practitioner

*Abbreviation:* HAM-D17, Hamilton depression scale (17-item version).

These measures were selected by a national multidisciplinary expert group appointed by scientific societies and professional organizations [17]. All measures reflected clinical services, which should be provided to all patients regardless of their individual characteristics, severity of disease, and so forth. However, some of the measures did not need to be performed if it could be demonstrated that they had recently been performed and documented elsewhere in the healthcare system. Others required a certain minimum length of stay to be relevant. Based on these criteria, the patients were individually classified as eligible or ineligible for the individual performance measures.

The quality of care was assessed both using the individual performance measure as well as by using two opportunity-based composite measures. Using the patient average method [18], one composite measure was constructed as a continuous variable of the total percentage of fulfilled eligible measures per individual. In addition, a dichotomous composite indicator was created with a cut-off of 70% or more of the eligible performance measures fulfilled as a measure of high quality of care. This cut-off is often used in analyses of composite indicators [18] and was a pragmatic estimate in which most of the care processes were fulfilled while still providing enough patients with the outcome to conduct meaningful analyses [19]. Additional cut-offs of 60 and 80% were applied in sensitivity analyses.

#### Clinical outcomes

Four clinical outcomes were investigated: (a) all-cause mortality was defined as death occurring up to 365 days after the day of hospital admission; (b) suicidal behavior was defined as poisoning (ICD-10: T36.0–T50.8), suicide or suicide attempts due to intentional self-harm (ICD-10: X60–X84 and Y870) up to 365 days after the day of hospital admission; (c) readmission for depression was defined as readmission within 365 days after discharge with a primary diagnosis of depression; and (d) all-cause readmission as any readmission within 365 days after discharge.

### Covariates

Relevant covariates were identified a priori using directed acyclic graphs and included age as a continuous variable and sex [20]. Models adjusting for these covariates constituted the primary analyses. However, socio-economic factors (SEF) could be considered potential confounding factors [21], although it is debated whether it is more correct to consider them as intermediate factors [22]. To explore the role of SEF, educational level, income, occupational status, and residency were included as covariates in a sensitivity analysis. Educational level was categorized in accordance with The International Standard Classification of Education (ISCED). Income was calculated as the average yearly total family income in the 5 years before admission. Employment status was classified into four categories: employed, public benefits, pensions, and students. Residency was registered based on home address and categorized in accordance with residency in one of the five administrative regions in Denmark.

### Statistical analyses

Analysis was first performed with all migrants compared with the Danish-born population and then performed with the two migrant subgroups (non-Western and Western) compared with the Danish-born population. To account for missing data, multiple imputation using chained equations was applied with missing data imputed 10 times using available patient characteristics including exposure and outcomes. Patients with missing exposure were excluded from the respective analysis.

The dichotomous composite score as well as the individual performance measures were examined using Poisson regression models with robust error variances, reporting relative risk (RR) with the corresponding 95% CI. The continuous composite score was examined using multivariable linear regression models, reporting percentage point difference (PPD) with corresponding 95% confidence intervals (CI).

The associations between migration status and clinical endpoints were determined using Cox proportional hazards regression analysis to compute hazard rate ratios (HR) for all-cause mortality and cause-specific HR (csHR) for suicidal behavior and readmissions presented with 95% CI. Administrative censoring at 365 days follow-up was applied. Unadjusted event proportions and adjusted risk differences (RDs) for the clinical endpoints were calculated from Aalen–Johansen cumulative incidence using bootstrap and death as a competing risk. Inverse probability of treatment weights was applied for adjusted analyses.

Analyses were performed using STATA v.16 (StataCorp LLC, College Station, TX) [23].

## Results

### Patient characteristics

Among the 20,508 patients admitted in the study period, 1,711 (8.4%) were migrants. Among these, 1,093 (63.8%) were from a non-Western country and 618 (36.2%) were from a Western country. The five most represented non-Western countries of origin were Turkey (*n* = 164), Iraq (*n* = 93), Iran (*n* = 93), Bosnia and Herzegovina (*n* = 85), and Afghanistan (*n* = 65). The five most represented Western countries were Poland (*n* = 99), Germany (*n* = 96), Sweden (*n* = 66), Norway (*n* = 59), and the United Kingdom (*n* = 56).

Compared to Danish-born patients, migrants were more likely to belong to the middle age groups (see Table 2). Migrants from Western countries were more likely to have a higher educational level than Danish-born patients and non-Western migrants, while the Danish-born patients had a higher income level than both migrant groups. Missing data on education were quite high among migrants. A larger proportion of Danish patients were retired, while especially migrants from non-Western countries were substantially more often unemployed. Both subgroups of migrants were more likely to live in the Capital Region than the Danish-born patients.

**Table 2.** Characteristics of patients admitted to hospital with depression (2011–2017).

	Total (*n* = 20,508)	Danish (*n* = 18,797)	Immigrant (all) (*n* = 1,711)	Non-Western countries (*n* = 1,093)	Western countries (*n* = 618)
Sex					
Woman	12,171 (59.4%)	11,147 (59.3%)	1,025 (59.9%)	638 (58.4%)	386 (62.5%)
Missing	0 (0%)	0 (0%)	0 (0%)	0 (0%)	0 (0%)
Age group					
0–25 years	2,426 (11.8%)	2,334 (12.4%)	92 (5.4%)	56 (5.1%)	36 (5.8%)
26–50 years	7,983 (38.9%)	6,984 (37.2%)	998 (58.4%)	730 (66.8%)	268 (43.4%)
51–75 years	7,655 (37.3%)	7,113 (37.8%)	542 (31.7%)	292 (26.7%)	250 (40.5%)
76+ years	2,445 (11.9%)	2,366 (12.6%)	79 (4.6%)	15 (1.4%)	64 (10.4%)
Missing	0 (0%)	0 (0%)	0 (0%)	0 (0%)	0 (0%)
Education					
Low	7,161 (34.9%)	6,709 (35.7%)	452 (26.4%)	348 (31.8%)	104 (16.8%)
Middle	8,239 (40.2%)	7,612 (40.5%)	627 (36.7%)	412 (37.7%)	215 (34.8%)
High	4,629 (22.6%)	4,186 (22.3%)	443 (25.9%)	216 (19.8%)	227 (36.7%)
Missing	479 (2.3%)	290 (1.5%)	189 (11.1%)	117 (10.7%)	72 (11.7%)
Income					
Low	6,822 (33.3%)	6,187 (32.9%)	635 (37.1%)	398 (36.4%)	237 (38.4%)
Middle	6,822 (33.3%)	6,192 (32.9%)	630 (36.8%)	413 (37.8%)	217 (35.1%)
High	6,822 (33.3%)	6,388 (34.0%)	434 (25.4%)	277 (25.3%)	157 (25.4%)
Missing	42 (0.2%)	30 (0.2%)	12 (0.7%)	5 (0.5%)	7 (1.1%)
Employment status					
Employed	7,603 (37.1%)	7,009 (37.3%)	594 (34.7%)	354 (32.4%)	240 (38.8%)
Unemployed	6,362 (31.0%)	5,538 (29.5%)	824 (48.2%)	625 (57.2%)	199 (32.2%)
Public pension	5,364 (26.2%)	5,173 (27.5%)	191 (11.2%)	49 (4.5%)	142 (23.0%)
Student	1,062 (5.2%)	988 (5.3%)	74 (4.3%)	46 (4.2%)	28 (4.5%)
Missing	117 (0.6%)	89 (0.5%)	28 (1.6%)	19 (1.7%)	9 (1.5%)
Regional residency					
Capital Region	6,991 (34.1%)	6,220 (33.1%)	771 (45.1%)	503 (46.0%)	268 (43.4%)
Region Zealand	2,859 (14.0%)	2,700 (14.4%)	159 (9.3%)	103 (9.4%)	56 (9.1%)
Region South	4,288 (20.9%)	4,008 (21.3%)	280 (16.4%)	167 (15.3%)	113 (18.3%)
Central Region	4,328 (21.1%)	3,994 (21.3%)	334 (19.5%)	233 (21.3%)	101 (16.3%)
Region North	1,859 (9.1%)	1,756 (9.3%)	103 (6.0%)	54 (4.9%)	49 (7.9%)
Missing	183 (0.9%)	119 (0.6%)	64 (3.7%)	33 (3.0%)	31 (5.0%)

### Quality of in-hospital care

**Figure 2.**
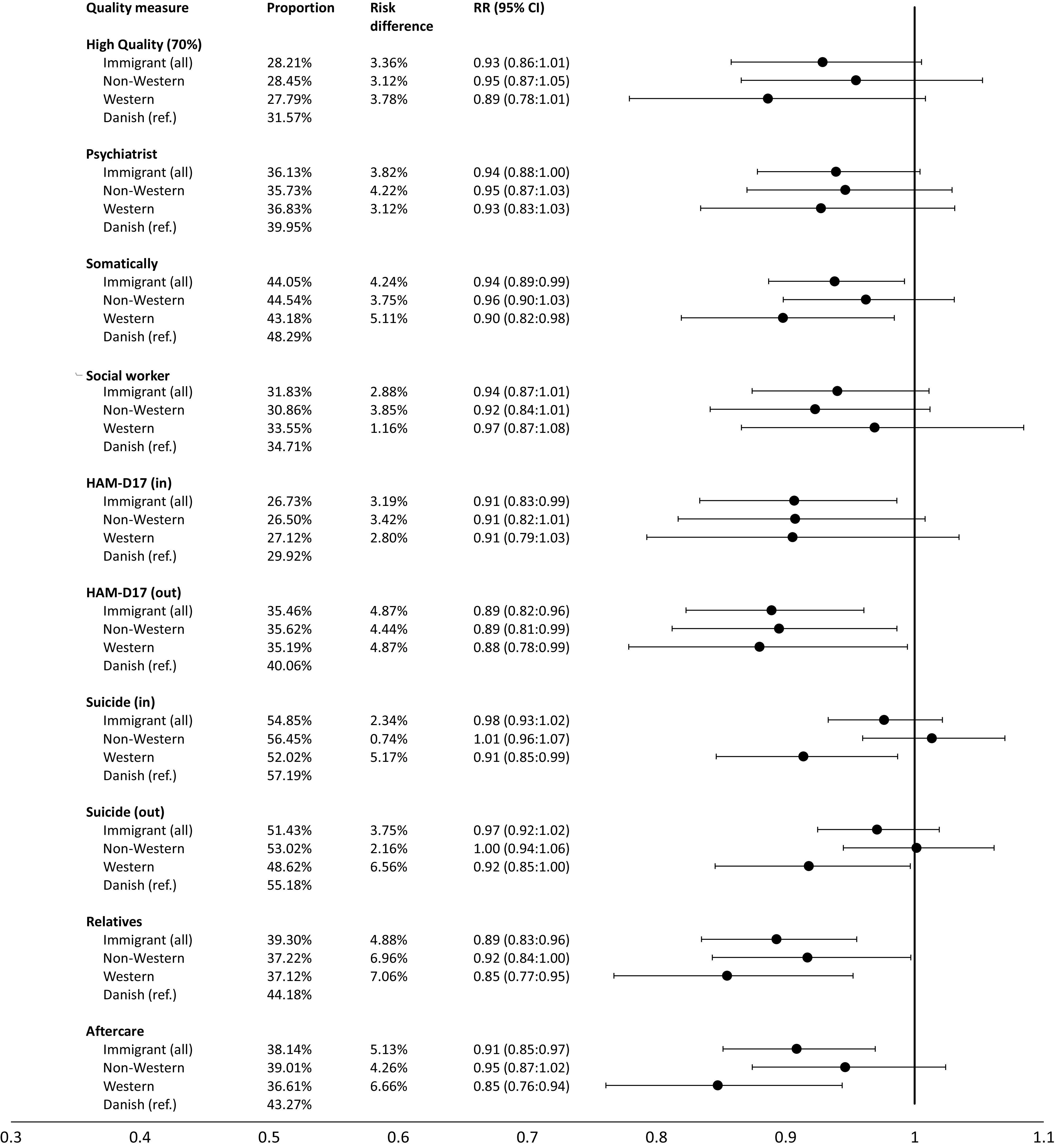
The association between migrant status and quality of care was measured as relative risk (RR, 95% CI) of fulfilling the composite performance measure (>70% fulfillment of eligible individual performance measures) and nine individual performance measures. Proportions of the migrant population who receive high quality and individual performance measures are provided (prop.) as well as the risk difference (risk dif.) from the reference group (Danish-born). The model was adjusted for sex and age.

Overall, 28.2% of the migrants received high quality of care defined by fulfilling 70% or more of the eligible performance measures. In comparison, the corresponding proportion was 31.6% for Danish patients. In the primary analyses, the RR for the 70% cut-off were 0.93 (95% CI 0.86:1.01) when comparing migrants to Danish-born patients (Figure 2). Sensitivity analyses with alternative cut-offs showed the same pattern for 60% (RR = 0.91, 95% CI 0.85:0.98) and 80% (RR = 0.96, 95% CI 0.90:1.00). Lower RR was also found for both migrant subgroups, however, the adjusted risk of receiving high quality was lower for the Western migrants (RR = 0.89, 95% CI 0.78:1.01) than non-Western migrants (RR = 0.95, 95% CI 0.87:1.05).

**Figure 3.**
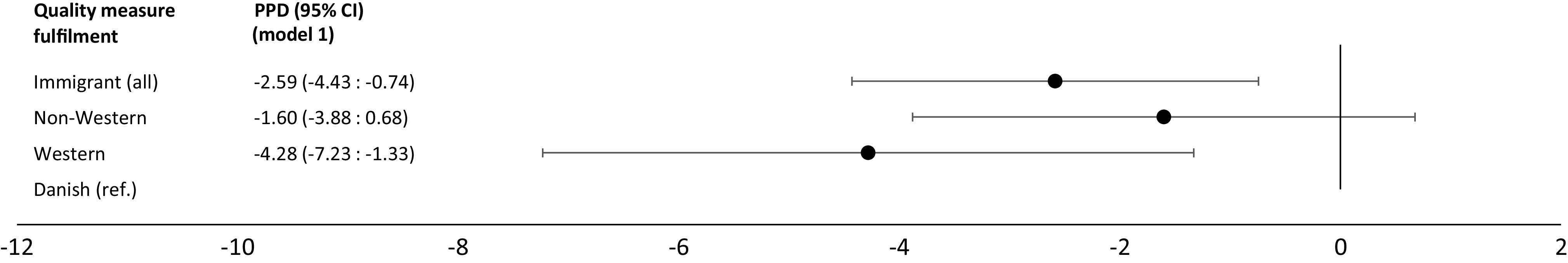
The association between migrant status and quality of care was measured as percentage point difference (PPD, 95% CI) in the overall quality measure fulfillment. The model was adjusted for sex and age.

Migrants received a reduced 2.59 PPD (95% CI −4.43: −0.74) in quality of care compared to Danish-born patients (Figure 3). Both subgroups received lower quality of care compared to Danish-born patients, however, the difference was only statistically significant for Western migrants.

A similar overall pattern was observed in the analyses of the individual performance measures, with migrant status being associated with a lower chance of fulfillment of all individual measures. The difference in proportions of the individual measures spanned from 5.13% (aftercare) to 2.34% (suicide in) with RRs ranging from 0.91 (95% CI 0.85:0.97) to 0.98 (95% CI 0.93:1.02) (Figure 2).

Supplementary analyses were performed with additional adjustments for SEF and residency (see Supplementary Material). In this analysis, the non-Western migrants generally received a quality that was equivalent or slightly better than the Danish-born patients.

### Clinical outcomes

**Figure 4.**
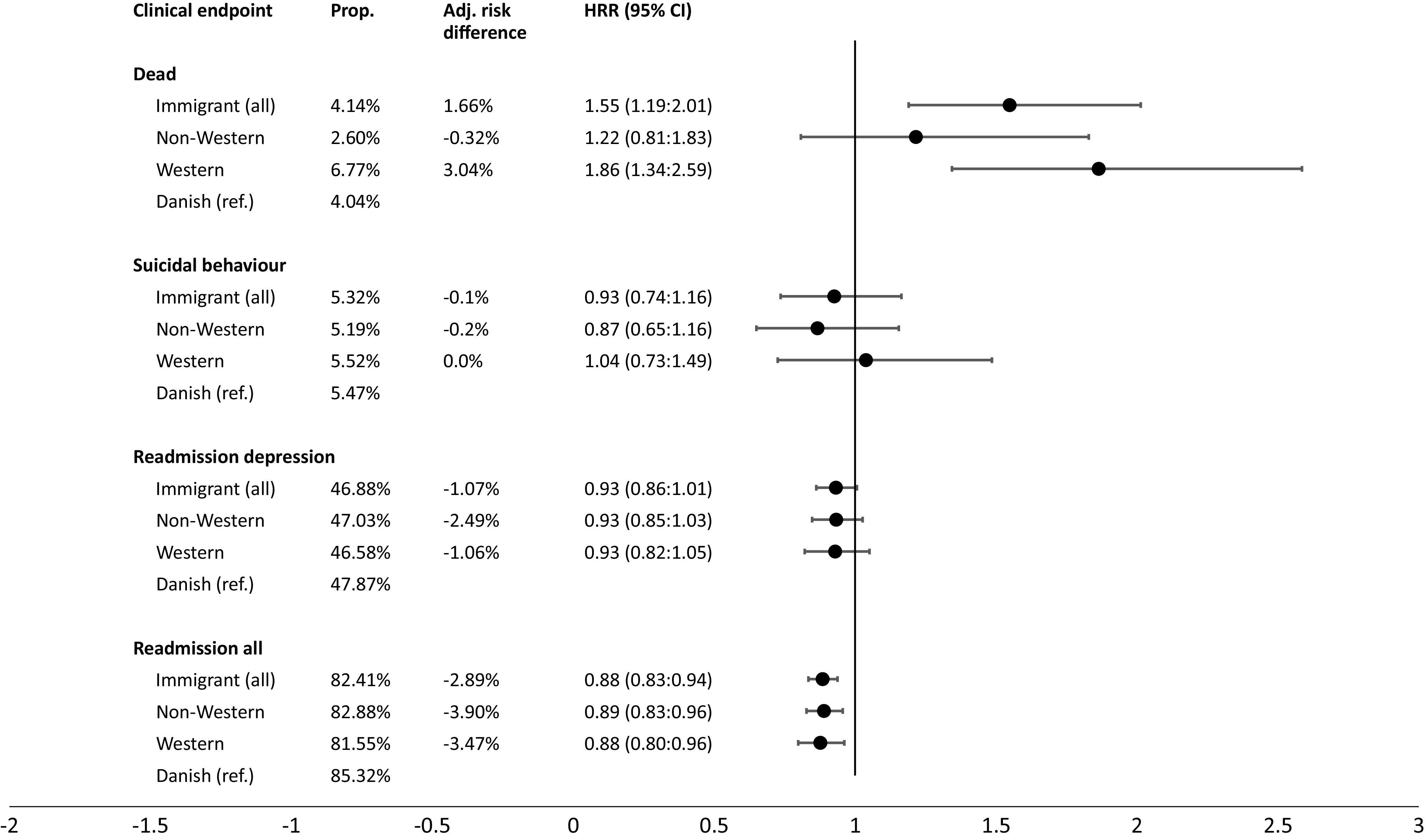
(Cause specific) Hazard rate ratio (HR, 95% CI) of the clinical endpoints: dead, suicidal behavior, depression-related readmission, and readmission, all at 1-year follow-up. Proportions (prop.) are provided as well as the adjusted risk difference (adj. risk dif.) from the reference group (Danish-born). The model was adjusted for sex and age.

The 1-year cumulative incidences of all-cause mortality were 4.14% among migrants and 4.04% among the Danish-born patients (Figure 4). However, there was 2.60% mortality among non-Western migrants and 6.77% among the Western migrants. The adjusted RD between migrants and Danes in 1-year all-cause mortality were 1.66%. However, based on subgroups, the adjusted RDs of all-cause mortality were −0.32% for migrants from non-Western countries and 3.04% for Western migrants. In the primary analyses, migrants had a significantly higher risk of 1-year all-cause mortality than Danish-born (HR = 1.55, 95% CI 1.19:2.01). Both migrant subgroups had an elevated risk of mortality, however, the HR was considerable higher and statistically significant for the Western migrants (HR = 1.86, 95% CI 1.34:2.59).

No clear association was found between being migrant status and suicidal behavior. The risks for all groups were approximately 5% in the 1-year follow-up period.

The absolute risk of 1-year all-cause readmission for all patient groups was above 80% and more than 45% were readmitted with depression. Migrant-status was associated with a lower depression (csHR = 0.93, 95% CI 0.86:1.01) and all-cause readmission rate (csHR = 0.88, 95% CI 0.83:0.94). The same associations were found for both migrant subgroups.

In the supplementary analyses, additional adjustments for SEF and residency had only a marginal impact on the results (see Supplementary Material).

## Discussion

### Principal findings

In this nationwide study of inpatients with MDD, a lower quality of in-hospital care was found among migrant patients compared with native Danes. Whereas the same pattern of lower quality of care was found for both migrant subgroups, non-Western migrants in general appeared to receive quality of care which was at least as high or higher than that of the Western migrants. Migrant patients, and particularly those from Western countries, had a higher all-cause mortality at 1-year follow-up. In addition, migrants had a lower risk of being readmitted for any reason as well as for MDD specifically. No difference in suicidal behavior was observed.

### Comparison with other studies

To the best of our knowledge, this study is the first to examine migrant status as a determinant of the quality of in-hospital depression care. Some studies have investigated migration-related differences in continuity of care among patients with depression, which indicate that migrants to a lesser degree adhere to antidepressants after discharge [24]. However, these care measures cannot rightly be said to only reflect the actions of the health care system [25].

Furthermore, no studies to our knowledge, have investigated migrant status as a determinant of mortality, suicide, or readmission among patients hospitalized with MDD.

### Differences in health

Although statistically significant and consistent, the between-group differences in the assessed quality of care between native Danes and migrants were moderate to small. However, taking all the findings in our study into account, a pattern emerges where migrants systematically received lower quality of care in combination with a worse clinical outcome in terms of higher all-cause mortality.

Readmissions are sometimes considered a measure of low quality of care [26]. However, the findings in this study, where migrants had a lower risk of readmission in combination with higher mortality, could indicate that in a welfare state with tax-financed universal health coverage, a lower readmission rate for vulnerable groups may reflect problems with continuity, compliance, and other barriers to access in case of relapse of depression or worsening of comorbidities. This adds to the emerging picture of systematic health differences between migrants and native Danes.

Several factors may play a role in the apparent difference in quality of care and clinical outcomes. First, cultural differences may involve perceptions of health and illness differently from the native population. However, this cultural approach seems to play a smaller role in this study, given the very diverse cultures represented in the migrant population. Second, the common element of being in a new country and the associated difficulties with navigating in an unknown health care system should be considered. Third, the difference could potentially be related to migrants being met in the new country with a different approach than the natives, for example, by discrimination [22]. Fourth, health care personnel may lack competencies when taking care of diverse populations with a migrant background due to a lack of relevant pre- and postgraduate training in diversity competencies [27].

These findings underpin the need for attention and interventions to ensure better access to care, quality of care, and clinical outcomes for migrant patients, for example, by ensuring inclusive and accessible promotion and prevention programs, strengthening mental health as part of general health services and ensuring timely diagnosis, treatment and rehabilitation of migrants, as recently suggested by the World Health Organization [2].

Further studies of the role of health care providers and health care systems in delivering high quality of care to all patients, regardless of their country of origin are also needed. These results warrant further investigations with increased sample size and follow-up time as well as qualitative studies on whether health care professionals’ conscious and unconscious biases may affect their ability to meet the needs of people of different ethnic backgrounds.

### Methodological strengths and limitations

Our study comes with both strengths and limitations. The study was population-based and involved all patients admitted for MMD in the Danish hospital system. Complete follow-up on the clinical outcomes was ensured using a unique personal identifier to enable linkage between public registries in which the coverage and validity of data are deemed to be high [12]. A limitation is that even though migrants were divided into two subgroups, the limited number of migrants made further subclassification difficult. This meant that ethnically heterogeneous groups were grouped together. Confounding is always an issue in observational studies. However, it has been debated how SEF should be treated in migrant studies [22]. If linguistic and cultural barriers or plain discrimination lowers the chances of getting an education or a job, being a migrant could lower one’s socioeconomic status. Socioeconomic position should then be considered an intermediate factor and statistically removing its influence will then render the effect of migration on health invisible [22]. For this reason, the model only adjusted for age and sex was chosen as the primary model. However, a sensitivity analysis including SEF and place of residence was performed. While non-Western migrants generally received a quality that was equivalent or slightly better than the Danish-born patients in this sensitivity analysis, the overall pattern of a migrant receiving worse quality and having more severe clinical outcomes remained, indicating that migrant status plays an independent role (see Supplementary Material).

### Generalizability

This study concerns patients hospitalized with MDD and can thus not necessarily be extrapolated to the entire population with depression, since most cases of mild depression are diagnosed and treated in the primary sector and many cases of moderate depression are treated as outpatients at the hospitals. The study was carried out in a universal healthcare system, which may limit the application of the results to other types of healthcare systems, for example, insurance-based systems such as in the United States or in countries with dissimilar migrant populations. The Scandinavian countries are often regarded as countries with free access to health care and low levels of inequality [28]. However, our findings from a relative egalitarian society could imply that the risks for migrant patients may be even greater in more unequal societies.

## Conclusion

Among inpatients with MDD in a universal tax-financed healthcare system, being a migrant was associated with a potential lower quality of in-hospital care and worse clinical outcomes. These results warrant further investigation on explanations for these inequalities, including whether health care professionals’ conscious and unconscious biases may affect their ability to meet the needs of people of different ethnic backgrounds. Furthermore, these findings underpin the need for attention and interventions to ensure better quality of care and clinical outcomes for migrant patients.

## Data Availability

Data cannot be shared publicly because of Danish legislation. Data can be accessed through the Danish Health Data Authority and Statistics Denmark for researchers at authorized institutions. Information on data access is available online (http://sundhedsdatastyrelsen.dk/da/forskerservice). Access to data requires approval from the Danish Data Protection Agency (https://www.datatilsynet.dk/english/legislation). The authors did not have special access privileges to these data.

## Supplementary material

For supplementary material accompanying this paper visit https://doi.org/10.1192/j.eurpsy.2022.2329. click here to view supplementary material
