# Supplementary material for: Differences in quality of care, mortality, suicidal behavior, and readmissions among migrants and Danish-born inpatients with major depressive disorder
Source: Eur Psychiatry. 2022 Oct 21;65(1):e69. doi: 10.1192/j.eurpsy.2022.2329 (PMC9677448; doi:10.1192/j.eurpsy.2022.2329)
Supplement: Supplementary file 1 [file S092493382202329Xsup001.docx]

**Supplementary material**


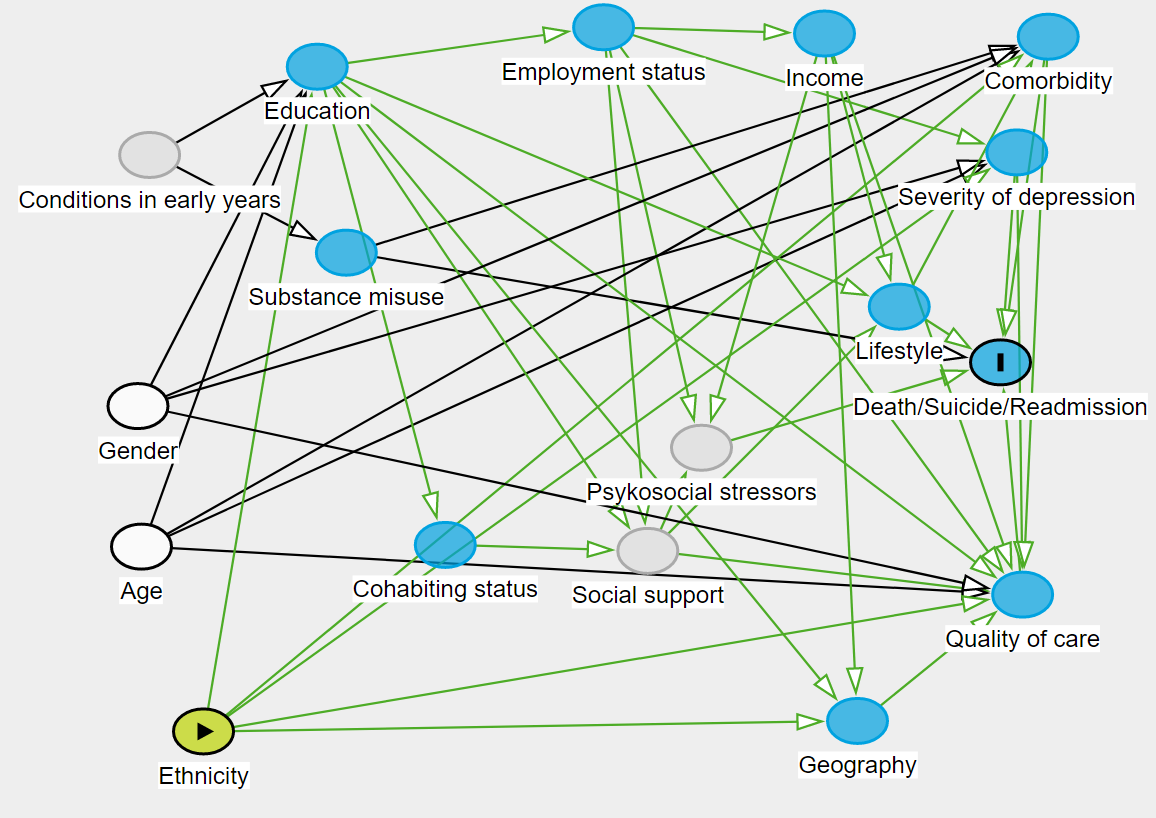

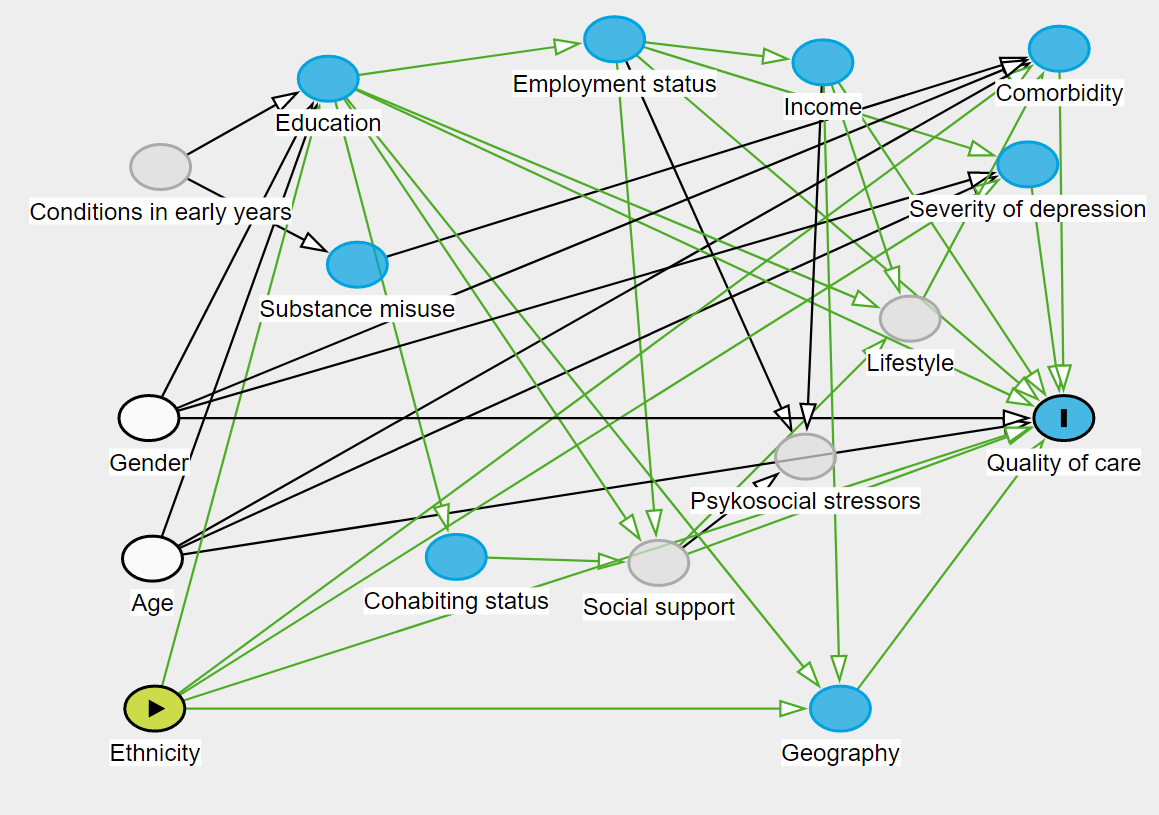
**Figure 1: Directed acyclic graphs depicting the relations between the potential variables**

**Figure 2: Fully adjusted models of associations between migrant status and quality of care**


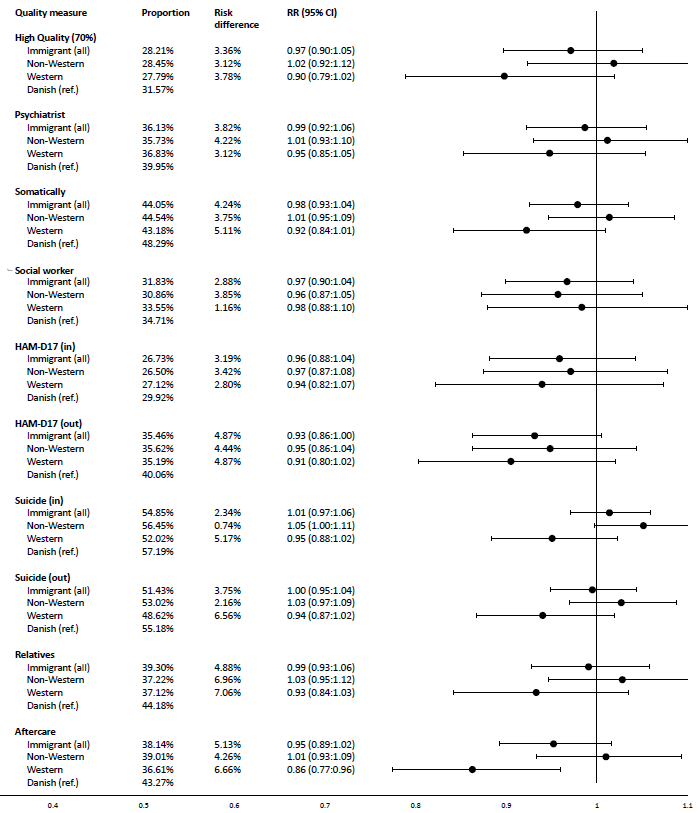


The association between migrant status and quality of care was measured as Relative Risk (RR, 95% CI) of fulfilling the composite performance measure (>70% fulfilment of eligible individual performance measures) and nine individual performance measures. Proportions of the migrant population who receive high quality and individual performance measures are provided (prop.) as well as the risk difference (risk dif.) from the reference group (Danish-born). The models were adjusted for sex, age, educational level, income, occupational status, and residency.

**Figure 3: Fully adjusted models of associations between migrant status and clinical outcomes**


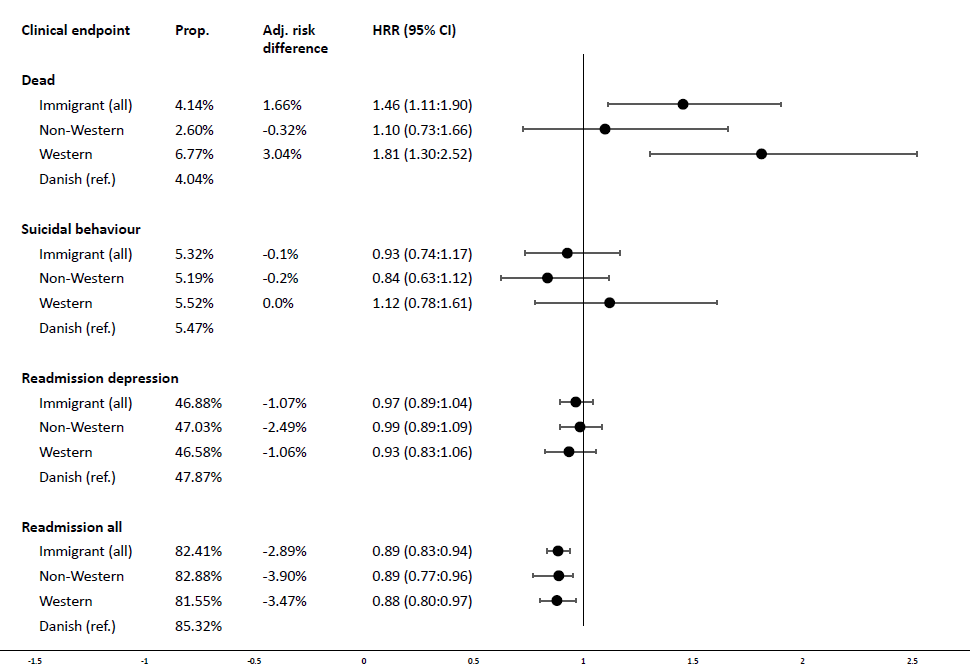


(Cause specific) Hazard Rate Ratio (HRR, 95% CI) of the clinical endpoints; dead, suicidal behaviour, depression-related readmission, and readmission, all at 1-year follow-up. Proportions (prop.) are provided as well as the adjusted risk difference (adj. risk dif.) from the reference group (Danish-born). The models were adjusted for sex, age, educational level, income, occupational status, and residency.
